# A Novel Abandoned Object Detection System Based on Three-Dimensional Image Information

**DOI:** 10.3390/s150306885

**Published:** 2015-03-23

**Authors:** Yiliang Zeng, Jinhui Lan, Bin Ran, Jing Gao, Jinlin Zou

**Affiliations:** 1 School of Automation and Electrical Engineering, University of Science and Technology Beijing, Beijing 100083, China; E-Mails: b20110370@xs.ustb.edu.cn (Y.Z); s20121029@xs.ustb.edu.cn (J.G.); s20130935@xs.ustb.edu.cn (J.Z.); 2 School of Transportation, Southeast University, Nanjing 210096, Jiangsu Province, China; E-Mail: bran@seu.edu.cn

**Keywords:** abandoned object detection, dual-background difference, three-dimensional information reconstruction, Road Traffic Surveillance

## Abstract

A new idea of an abandoned object detection system for road traffic surveillance systems based on three-dimensional image information is proposed in this paper to prevent traffic accidents. A novel Binocular Information Reconstruction and Recognition (BIRR) algorithm is presented to implement the new idea. As initial detection, suspected abandoned objects are detected by the proposed static foreground region segmentation algorithm based on surveillance video from a monocular camera. After detection of suspected abandoned objects, three-dimensional (3D) information of the suspected abandoned object is reconstructed by the proposed theory about 3D object information reconstruction with images from a binocular camera. To determine whether the detected object is hazardous to normal road traffic, road plane equation and height of suspected-abandoned object are calculated based on the three-dimensional information. Experimental results show that this system implements fast detection of abandoned objects and this abandoned object system can be used for road traffic monitoring and public area surveillance.

## 1. Introduction

With the rapid development of economy and trade, the number of road vehicles is increasing very fast. In recent years this has led to the frequent appearance of road abandoned objects on roads. Traditional transport facilities cannot meet the need of modern society to detect road abandoned objects in time. Abandoned objects not only reduce the efficiency of transportation, but also cause potential safety problems to all traffic. Once an abandoned object is in the road, it may lead to economic losses and even cause loss of life, so it is very important to handle abandoned objects efficiently and in time.

**Figure 1:**
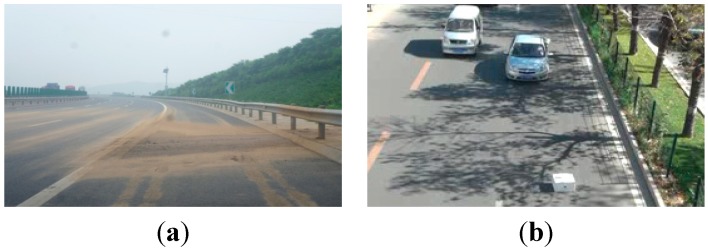
General abandoned objects in road traffic. (**a**) Harmless abandoned object; and (**b**) Hazardous abandoned object.

There are different kinds of abandoned objects on roads, but not all of them threaten traffic order and safety, so road abandoned objects can be classified into harmless abandoned objects and hazardous abandoned objects according to the threat they pose to traffic safety. Harmless abandoned objects are abandoned objects which cannot threaten traffic safety or order. Hazardous abandoned objects are abandoned objects which can threaten to traffic safety and normal traffic order. To prevent traffic accidents with high efficiency, it is very important to evaluate the danger of abandoned objects through an efficient and accurate method. General abandoned objects are shown in Figure 1. Figure 1a shows a harmless abandoned object (scattered sand) in the road, and Figure 1b shows a hazardous abandoned object (a box) in the road.

Abandoned objects may be detected by different methods, but video-based abandoned objects detection methods have more advantages than other methods, such as low cost, and being easy to mount and repair. Currently, video surveillance systems are already able to collect traffic data, detect traffic congestion and traffic accidents, but few video surveillance systems are used for abandoned object detection. Most of abandoned object detection systems are used for detection of abandoned objects in public areas such as parks, train stations and airports. For example, abandoned object detection systems are used in public areas in [1,2,3,4] and also used for public security [5]. In [6], an abandoned object detection algorithm is proposed specially for the highway traffic scenario. The authors try to detect abandoned objects using a Gaussian mixture model and decrease the influence of the noise. However, their method cannot acquire the height information of the object, which is one of the key parameters of hazardous abandoned objects. A video-based abandoned object detection method proposed in [7], although it can implement real-time detection of non-flat abandoned object using a moving camera and reduce the false alarms caused by shadows and rain, it is not for road traffic monitoring systems and fails in detecting flat objects. A real time abandoned object detection and owner recognition algorithm is proposed in [8]. This detection algorithm can be used for public area surveillance and road traffic monitoring, but it cannot select the hazardous objects. In [9], a robust abandoned objects detection algorithm based on the mixture of the Gaussian background subtraction and foreground analysis is proposed. This algorithm can eliminate noises from walking people, waving trees, and so on, but it cannot evaluate the danger of abandoned objects and the accuracy of abandoned object detection is influenced by adverse weather conditions and low contrast situations. A double-background abandoned object detection algorithm is introduced in [10]. This method can be used in all kinds of public areas and reduces the false alarms caused by illumination changes or low quality video, but it provides no further information about abandoned objects, so it also cannot evaluate the danger of an object. A multi-camera-based abandoned object detection algorithm is proposed in [11]. Although it can minimize the effect of noise from light, position and occlusion, it is time consuming because of its 3D object tracking. Overall, most of the previous abandoned object detection systems are based on the use of one camera. However, these methods cannot evaluate the danger of the abandoned objects such as providing the height parameters of an object. Using multiple cameras with overlapping fields of view can cope with occlusions of various types better. Therefore, it needs to propose an efficient method with multiple cameras to obtain the 3D image information for road abandoned object detection.

In most intelligent transportation visual monitoring systems, the monitoring cameras are installed spread in different areas to cover more geographic monitoring regions. These systems have been widely applied to moving target detecting and tracking, traffic flow detection, traffic condition assessment, Red Light Runners detection, reverse driving detection and so on. They usually use monocular visual information, which is two-dimensional video surveillance, to obtain and analyze the traffic parameters. However, there are still some limitations in this road monitoring pattern. In recognition and tracking of traffic targets, it is difficult to get enough target features from a single visual angle, especially under circumstances of severe occlusion or appearance changes. In addition, with a monocular camera it is harder to obtain the 3-D depth information, which is very useful for target recognition and location. Therefore, in order to improve the accuracy in different applications (such as target recognition and location, road infrastructure analysis, abandoned objects detection *et al.*), it is warranted to have multiple cameras covering overlapping regions for intelligent transportation visual monitoring systems.

**Figure 2:**
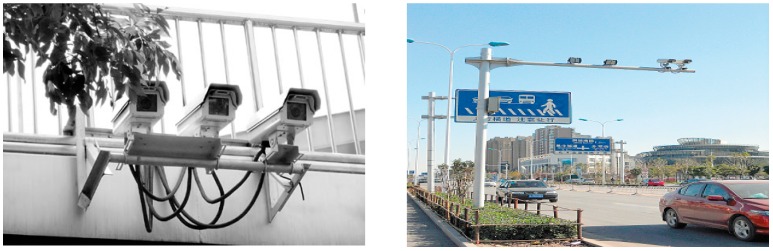
Multi-cameras road monitoring systems.

Because the 3-D reconstruction technique based on multiple cameras with overlapped view has not been applied in the traffic monitoring systems, it is difficult to objectively argue for the return on investment between multiple cameras with overlapped view and multiple cameras with more coverage. From another point of view, multiple cameras with overlapped view can effectively solve the existing problems of target occlusion, light sensitivity and limited applications. It is an important supplement to the practical scenarios where cameras are spread to cover a larger geographic region. Meanwhile, the price of cameras is cheaper than ever, and there are lots of road cameras with overlapping coverage, which have been installed as shown in Figure 2, in urban traffic area in China. These provide an infrastructure guarantee for abandoned object detection in multiple cameras road monitoring systems. When the multiple cameras with overlapping coverage are installed in accident-prone areas, it is helpful for reducing the incidence of traffic accidents and boosting the return on investment.

**Figure 3:**
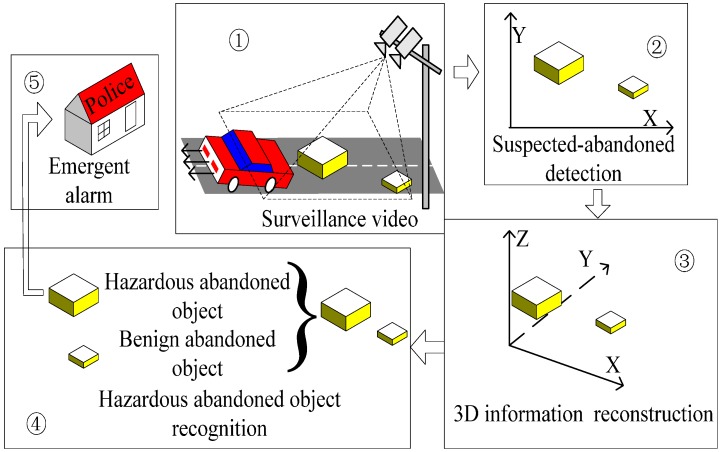
Sketch map of BIRR.

Therefore, a 3D image information-based abandoned object detection idea is proposed in this paper. This new idea is implemented by the proposed BIRR algorithm. A sketch map of BIRR is shown in Figure 3.

**Figure 4:**
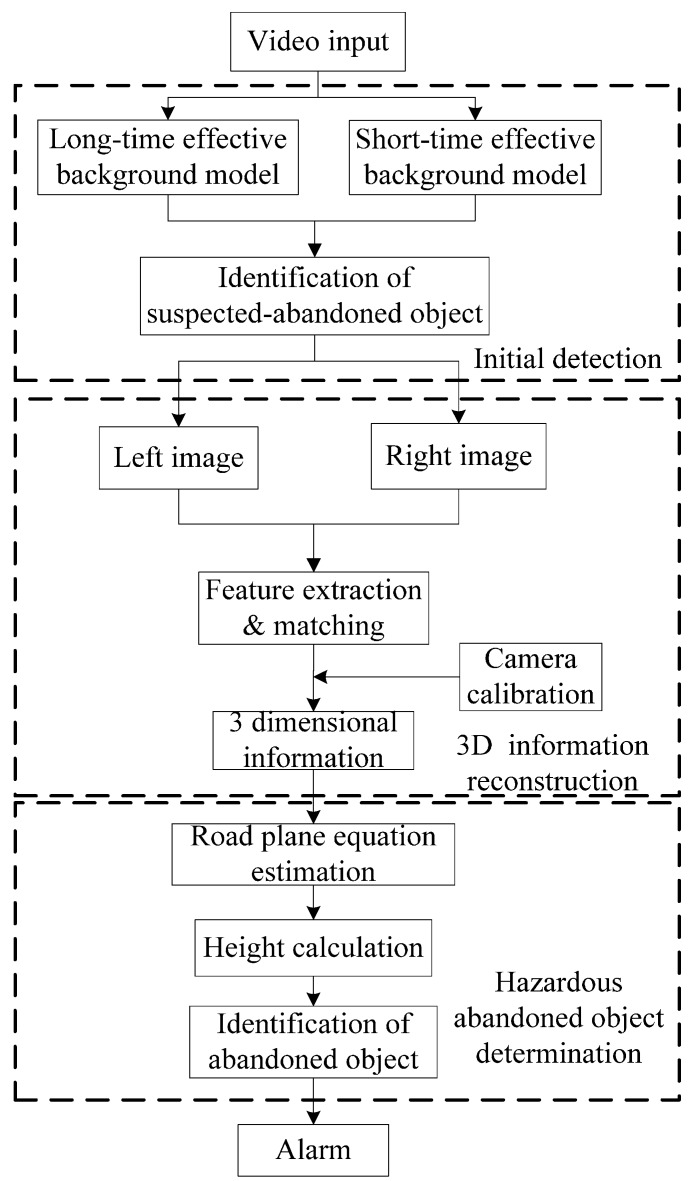
Block diagram of the abandoned object recognition system.

In this BIRR algorithm, suspected abandoned objects are detected by a static foreground region segmentation algorithm. After a suspected abandoned object is confirmed, its 3D information is reconstructed by the proposed algorithm. Finally, hazardous abandoned objects are recognized and alarm information sent to the system administrator. The whole BIRR mainly consists of three concepts: initial detection, 3D information reconstruction and hazardous abandoned object recognition. The overall block diagram is shown in Figure 4.

This paper is an expansion and elaboration of a previous Transportation Research Board conference paper [12]. Unlike the former conference paper that only brief describes the original algorithm, without delving into the details of the method and examples, these are presented here, and some improved algorithms are also proposed to improve the abandoned object detection system.

## 2. Methods and Theories

### 2.1. Static Foreground Region Segmentation 

In traditional detection, the temporary-background difference method is used to detect static objects for surveillance systems [13,14]. It has a two layer background. The first layer is to store the original background, which is established *a priori*. The second layer stores the updated background. In order to adapt to complex environments, backgrounds will be re-established at regular intervals. The new background becomes the current background and the old background becomes the previous background at the same time. Because of background updating, the suspected abandoned object will have blended into the current background after updating the background for a while, thus static objects can be calculated by seeing the difference between the first layer background and the second layer background. The temporary-background difference method can effectively detect static foreground objects, but holes in the object region and discrete noise will affect the detection accuracy when the target size is small. Meanwhile, stationary vehicles and people may be regarded as abandoned objects, so a novel static foreground object segmentation method based on a dual-background difference algorithm is proposed for road surveillance in this paper. The short-term background model and long-term background model are established first, and then the static foreground region will be obtained through the above two models. Finally, the static foreground region is regarded as a suspected abandoned object.

A. Short-term background modeling and updating

**Figure 5:**
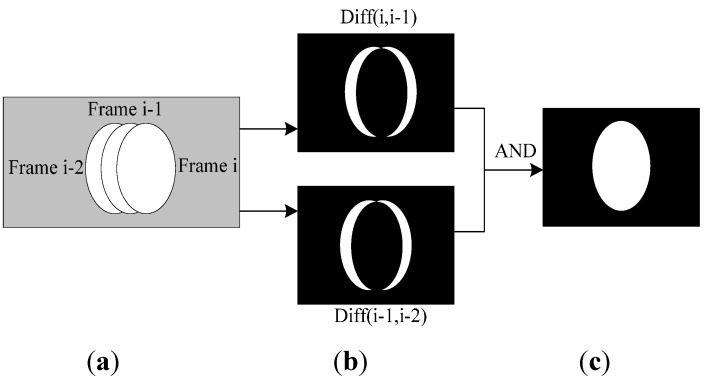
Binary motion region extraction. (**a**) motion region in three sequent frames; (**b**) binary difference image; (**c**) Binary motion region extraction result.

The short-term background model has a faster updating rate. It makes stationary targets blend into the background in a short time due to this fast updating rate. The traditional Surendra algorithm can acquire the background quickly, but it does not perform further processing of frame differences [15]. This existing problem may cause a cross-regional foreground with similar texture to be regarded as background. In addition, the extracted region of motion is often greater than the actual region, which leads to detection inaccuracy. In order to solve this problem, an improved Surendra background molding algorithm is proposed in this paper to establish the short-term background. The algorithm calculates the motion region based on a three frame difference. It keeps background points unchanged in the motion region and updates background points using the current and former two frames in the non-motion region, so background images can be extracted after a period of time. The algorithm can be divided into the following steps:

(1) Define the first image $I_{0}$ as background image $B_{0}$;
(2) Set the number of iterations as N;
(3) Get binary difference image between current frame and previous frame:
  (1) $$G_{i,i-1}=\left\{ \begin{array}{cc} 1 & if\left|I_{i}-I_{i-1}\right|>T_{0}\\ 0 & else \end{array}\right.$$
  and the binary difference image of former two frames is:
  (2) $$G_{i-1,i-2}=\left\{ \begin{array}{cc} 1 & if\left|I_{i-1}-I_{i-2}\right|>T_{1}\\ 0 & else \end{array}\right.$$
  where $I_{i}$ is the current frame and $I_{i-1}$, $I_{i-2}$ are the former two frames; $\left|I_{i}-I_{i-1}\right|$ is the difference image between two consecutive frames; $T_{0}$ and $T_{1}$ are thresholds for binarization.
(4) Obtain the binary motion region edge by using the ‘AND’ operation
  (3) $$G_{i1}=G_{i,i-1}\&G_{i-1,i-2}$$
  and background subtraction method [16] shown as follow is adopted to fill the region:
  (4) $$G_{i}=\{(x,y):\left|I_{i}(x,y)-B_{i-1}(x,y)\right|>T_{2},\text{ (}x,y)\in G_{i1}\}$$
  where $B_{i-1}(x,y)$ represents the current background image of the binary motion region. The binary motion region extraction is shown in Figure 5.

(5) Update the instant background $TB_{i}$ by binary motion region image $G_{i}$ as follows:
  (5) $$TB_{i}(x,y)=\{\begin{array}{cc} I_{i}(x,y) & if\;G_{i}(x,y)=0\\ B_{i-1} & if\;G_{i}(x,y)=1 \end{array}$$

Then, the new background $B_{i}(x,y)$ will be updated by the following formula:

(6) $$B_{i}=\varepsilon \times TB_{i}+(1-\varepsilon )\times B_{i-1}$$

where ε is the update rate.

(6) Let i=i+1, return to step 3 and iterate. The iteration will finish when i=N, then $B_{i}$ is regarded as extracted background.

This algorithm can quickly adapt to real-time changes in the background image, and especially adapt to changes of light. It does not need to initialize the background, and background image can be quickly extracted by iteration. The background has a fast update rate, so the static objects can blend into the background quickly.

B. Long-term background modeling and updating

This paper selects an improved Gaussian mixture background model [17] as the long-term background model. The speed of background updating can be controlled by the learning rate. Because the traditional Gaussian mixture background model uses a fixed K Gaussian distribution to describe every pixel, some pixels cannot be described accurately. Meanwhile, traditional background update algorithms lead to non-convergence problems. To make up for these shortcomings, this paper presents an improved Gaussian mixture model which is based on a Gaussian mixture distribution model of adaptive distribution. It adjusts the distribution numbers of pixel models according to the actual situation and updates models by improved L-Windows [18]:

(1) Distributed parameter model updating

Traditional L-Windows rules may cause the non-convergence, so an improved L-Windows method is appropriate for the situation where the number of samples is less than constant L. When the number of sample video images is less than L, the following update equations are used:

(7) $$w_{k,t}=w_{k,t-1}+\frac{1}{\sum_{i=1}^{K}ms_{i}+1}(m_{k}-w_{k,t-1})$$

(8) $$\mu_{k,t}=\mu_{k,t-1}+\frac{m_{k}}{ms_{k}}({Χ}_{t}-\mu_{k,t-1})$$

(9) $$\sum_{k,t}=\sum_{k,t-1}+\frac{m_{k}}{ms_{k}}(({Χ}_{t}-\mu_{k,t-1}){\left({Χ}_{t}-\mu_{k,t-1}\right)}^{T}-\sum_{k,t-1})$$

where $w_{k,t}$ is the weight parameter of the *k* th Gaussian component at time *t*, μ is the mean, $ms_{k}$ represents the total number of pixels that match with k th Gaussian model, and it reflects the learning rate of Gaussian model.

When the number of sample video images is greater than or equal to L, update equations are described as follows:

(10) $$w_{k,t}=w_{k,t-1}+\alpha (m_{k}-w_{k,t-1})$$

(11) $$\rho_{k}=m_{k}(\frac{1-\alpha }{ms_{k}}+\alpha )$$

(12) $$\mu_{k,t}=\mu_{k,t-1}+\rho_{k}({Χ}_{t}-\mu_{k,t-1})$$

(13) $$\sum_{k,t}=\sum_{k,t-1}+\rho_{k}(({Χ}_{t}-\mu_{k,t-1}){\left({Χ}_{t}-\mu_{k,t-1}\right)}^{T}-\sum_{k,t-1})$$

(14) $$ms_{k}(t)=\sum_{i=1}^{t}m_{k}(i)$$

where α and ρ are learning rate.

The value of $m_{k}$ is 1 when ${Χ}_{t}$ match with the kth Gaussian model. Otherwise, it is 0. The method is to learn independently for each Gaussian model distribution. It will not only make Gaussian mixture model converge faster, but do also lay foundation to the later stages of object detection.

(2) Adaptive increasing and discarding of distribution number

Adaptive increasing and discarding of the distribution number is an effective way to solve the problem that fixed distribution cannot accurately describe changes in the complex traffic environment. If the video sample *x_j_* cannot find any distribution to match with it, it means that original background model cannot accurately describe the actual changes of the traffic environment, then a new distribution *C_k_* needs to be generated, and the initial parameter value is set as $\mu_{k}=x_{j}$. According to experience, *w_k_* takes a smaller value, *σ_k_* takes greater value, but some new distributions which may be caused by the noise of the camera or a particular gray level of objects can match with few new samples. In order to avoid having too many distribution numbers, some distributions must be removed. The strategy is that all of the current distributions are checked with M frame images per check. If the weight *w_k_* of a distribution *C_k_* is less than 1/*M*, the distribution will be discarded. It will not only realize adaptive increasing and discarding of the distribution numbers of the model but will also makes model adapt to changes in the traffic environment.

C. Dual-background difference algorithm

**Figure 6:**
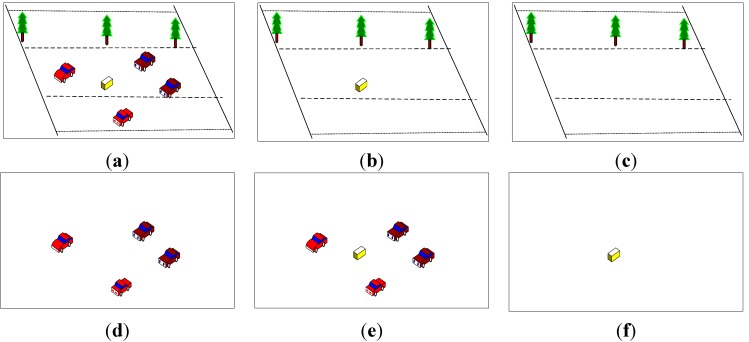
Detection method based on double background models. (**a**) Current image; (**b**) Short-term background image; (**c**) Long-term background image; (**d**) Short-term foreground image; (**e**) Long-term foreground image; and (**f**) Abandoned object.

Abandoned objects have the characteristic of blending into the background gradually because of the background updating process. Road abandoned objects do not blend into both long-term and short-term background because of their speed at first, but gradually blend into the short-term background after staying still on the scene for a while. The detection method based on double background models is shown in Figure 6.

**Figure 7:**
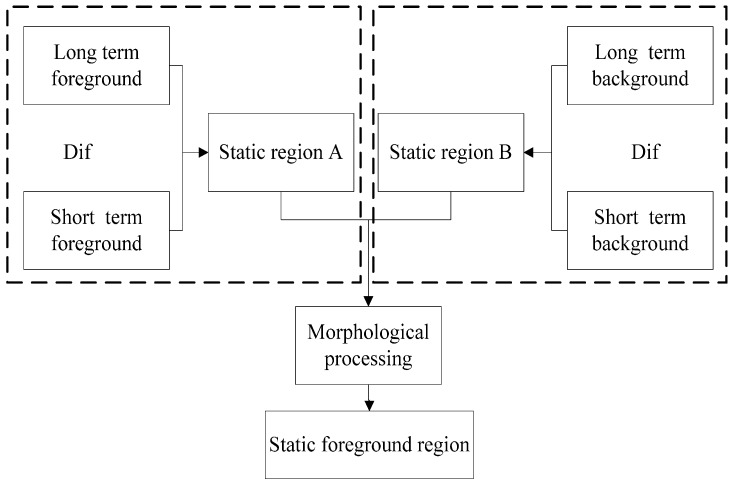
Static foreground region segmentation.

The static foreground region can be isolated from the difference between (d) and (e), as shown in Figure 6, but due to the different background models, the detected moving foreground region may not completely overlap, so the difference between above two foreground images not only contains a static foreground region, but also the contour of moving objects. This paper combines the difference between (d) and (e) with the difference between (b) and (c) to obtain the static foreground region. The segmentation process of static foreground region is shown in Figure 7. As shown in Figure 7, the short-term foreground and long-term foreground are obtained from the difference between two background models. Abandoned objects have been blending into the short-term foreground but not long-term background after leaving them on the road for some time. Thus, the short-term foreground contains the moving object region, while the long-term foreground contains the moving object region and abandoned object region.

Here, we re-defined the short-term background as $BG_{short}$, and the long-term background as $BG_{long}$. Then, the short-term foreground and long-term foreground can be respectively represented by the following formulas:

(15) $$FG_{short}=\{\begin{array}{cc} 1 & \left|I_{t}(x,y)-BG_{short}(x,y)\right|>T_{S}\\ 0 & else \end{array}$$

(16) $$FG_{long}=\{\begin{array}{cc} 1 & \left|I_{t}(x,y)-BG_{long}(x,y)\right|>T_{L}\\ 0 & else \end{array}$$

where $T_{s}$ and $T_{L}$ are the threshold for short-term background model and long-term background model, respectively.

By performing an XOR operation between the above two foreground images one could obtain the static foreground A (dual-foreground segmentation image); the operation formula is as follows:

(17) $$FG_{A}=FG_{short}\;XOR\;FG_{long}=FG_{short}^{\prime }FG_{long}+FG_{short}FG_{long}^{\prime }$$

The abandoned object has been integrated into the short-term background, but not into the long-term background at this point, so the difference between the short-term and long-term background models can obtain the static foreground region B (dual-background segmentation image):

(18) $$FG_{B}=\{\begin{array}{cc} 1 & \left|BG_{long}(x,y)-BG_{short}(x,y)\right|>T_{B}\\ 0 & else \end{array}$$

Finally, the static foreground region (suspected abandoned object) is obtained by an AND operation between region A and region B. Noise from complex environments and light can be removed by morphological filtering. Because the static foreground region needs further processing to identify it as a hazardous abandoned object, it is defined as a suspected abandoned object.

### 2.2. Three-Dimensional Information Reconstruction

The hazardous abandoned object recognition criterion is based on 3D information. 3D information reconstruction is important to all detection research [19,20]. To reconstruct 3D information, camera internal and external parameters should be acquired first [21].

(1) Getting camera parameters

To obtain camera internal parameter for 3D information reconstruction, a plane calibration method is used in this paper. This method is originally proposed by Zhang [22], and it has advantages of both traditional calibration methods and self-calibration methods. The external parameters R and t are derived from a pair of images based on Scale Invariant Feature Transform (SIFT) feature matching [23]. It is beneficial to decrease the errors of 3D information reconstruction.

Digital cameras are used to take pictures of objects at two different locations, and the object surface is reconstructed by using the matching results of these two pictures. If pictures are taken at location A and B, the camera coordinate system of position A is taken as the world coordinate system. Then the extrinsic parameter matrix of the camera at position A will be [I|0]; the projection matrix of A position will be $P_{A}=K_{1}\left[I|0\right]$; the projection matrix of B position will be $P_{B}=K_{2}\left[R|t\right]$ where I is the three dimensional unit matrix, K is the camera intrinsic parameter, R and t represent the relative rotation matrix and translation vector from position A to position B. K is obtained using Zhang’s calibration method. Because $P_{A}$ is known, $P_{B}$ can be calculated as long as R and t are acquired. Then the 3D point coordinates of object surfaces could be obtained by trigonometry.

If the homogeneous coordinates of any pair of match points $m_{1}\in I_{1}$, $m_{2}\in I_{2}$ are $m_{1}={\left(x_{1},y_{1},1\right)}^{T}$ and $m_{2}={\left(x_{2},y_{2},1\right)}^{T}$, the relations of prospective projection are $\lambda_{1}m_{1}=K_{1}\left[I|0\right]M$ and $\lambda_{2}m_{2}=K_{2}\left[R|t\right]M$.

While $\lambda_{1}$, $\lambda_{2}$ are constant factors, M is the homogeneous coordinates of key points. After removing $\lambda_{1}$ and $\lambda_{2}$, ${\left(m_{2}\right)}^{T}Fm=0$ can be obtain. Here, F is fundamental matrix and $F=K_{2}^{-1}[t]\times RK_{1}^{-1}$. [t]× is anti-symmetric matrix of vector t. If $t={\left(t_{x},t_{y},t_{z}\right)}^{T}$, [t]× is as follows:

(19) $$[t]\times =\left[\begin{array}{ccc} 0 & -t_{x} & t_{y}\\ t_{x} & 0 & -t_{x}\\ -t_{y} & t_{x} & 0 \end{array}\right]$$

The essential matrix is defined as E=t×R=[t]×R, then there is $E=K_{2}^{T}FK$. Because of environment noise in reality, the essential matrix needs to be modified with further processing. In fact, the essential matrix E is estimated and modified by a least squares approximation. In this process, singular value decomposition is carried out for essential matrix E first. Then the diagonal matrix is D=diag(r,s,t), and there is r≥s≥t. If we let k=(r+s)/2, the diagonal matrix can be obtained as diag(k,k,0). The least squares approximation of essential matrix for E is E′=Udiag(k,k,0)V. Then, singular value decomposition will be carried out for matrix E′. Two unitary matrices U and V of 3rd order and diagonal matrix S of 3rd order is obtained as a result $E\prime =USV^{T}$.

The rotation matrix R and translation vector t can be represented respectively as $R=UWV^{T}$, $R=UW^{T}V^{T}$, $t=u_{3}$ or $t=-u_{3}$ where $u_{3}$ is the last row of matrix U and $W=\left[{\left(\begin{array}{ccc} 0 & 1 & 0 \end{array}\right)}^{T}{\left(\begin{array}{ccc} -1 & 0 & 0 \end{array}\right)}^{T}{\left(\begin{array}{ccc} 0 & 0 & 1 \end{array}\right)}^{T}\right]$.

Because there are two possible values for each R and t, the projection matrix $P_{B}$ may have four values. They are $K[UWV^{T}|u_{3}]$, $K[UWV^{T}|-u_{3}]$, $K[UW^{T}V^{T}|u_{3}]$, $K[UW^{T}V^{T}|-u_{3}]$. To confirm the values of R and t, 3D points coordinates are calculated by several pairs of points in images at first. Then, according to depth values of 3D points in two cameras, *R* and *t* are selected. It is the right value of R and t when the depth values of 3D points are positive. 3D coordinates of object points can be calculated after acquiring camera parameters.

(2) Calculating 3D coordinates of points

The optical center of a camera at position A is selected as the world coordinate system and the 3D coordinates of matching points are calculated by using the projection matrix $P_{A}=K_{1}\left[I|0\right]$ and $P_{B}=K_{2}\left[R|t\right]$. Now there is a pair of matching points that are $m_{1}={\left(u_{1},v_{1},1\right)}^{T}$ and $m_{2}={\left(u_{2},v_{2},1\right)}^{T}$. The corresponding 3D homogeneous coordinates are $M={\left(X,Y,Z,1\right)}^{T}$, so that $m_{1}=k_{1}P_{A}M$ and $m_{2}=k_{2}P_{B}M$, where $k_{1},k_{2}$ are proportional coefficients. After removing the proportional coefficient, the following formula can be obtained:

(20) $$\begin{array}{l} (u_{1}m_{31}^{1}-m_{11}^{1})X+(u_{1}m_{32}^{1}-m_{12}^{1})Y+(u_{1}m_{33}^{1}-m_{13}^{1})Z=m_{14}^{1}-u_{1}m_{34}^{1}\\ (v_{1}m_{31}^{1}-m_{21}^{1})X+(v_{1}m_{32}^{1}-m_{22}^{1})Y+(v_{1}m_{33}^{1}-m_{23}^{1})Z=m_{24}^{1}-v_{1}m_{34}^{1}\\ (u_{2}m_{31}^{2}-m_{11}^{2})X+(u_{2}m_{32}^{2}-m_{12}^{2})Y+(u_{2}m_{33}^{2}-m_{13}^{2})Z=m_{14}^{2}-u_{1}m_{34}^{2}\\ (v_{2}m_{31}^{2}-m_{11}^{2})X+(v_{2}m_{32}^{2}-m_{22}^{2})Y+(v_{2}m_{33}^{2}-m_{23}^{2})Z=m_{24}^{2}-v_{1}m_{34}^{2} \end{array}$$

The above four simultaneous equations are about three variable of X, Y and Z, so the minimum value of X, Y and Z can be solved from above equations.

### 2.3. Hazardous Abandoned Object Recognition

The road abandoned objects mentioned in this paper are of different types such as illegally stopped vehicles, abandoned boxes and so on. Because of the abandoned object’s similar shape and complex types, two-dimensional image information can only judge whether abandoned objects exist on road, but it cannot evaluate the danger they may pose. Therefore, the final detection result may cause unnecessary alarm. To determine the potential danger of abandoned objects, this paper calculates the maximum height of suspected abandoned object by 3D information through steps of road plane estimation and hazardous abandoned object recognition. After recognizing hazardous abandoned objects, a warning alarm is sent to the traffic management department based on the final classification results. The alarm has great meaning to traffic managers who can take reasonable emergency measures based on the alarm information for eliminating hazardous abandoned objects from the road. The two key procedures are introduced in detail as follows:

(1) Road plane equation extraction

The abandoned object’s height is relative to the road plane. Therefore, we need to calculate the road plane equation. Most of vision-based road plane extraction algorithms are feature-based recognition algorithms [24], which are less effective when an obstacle’s color is nearly same or the same as the road color, so his paper uses the RANSAC algorithm to obtain the current most probable road plane equation from images using 3D information. Many experiments show that parameter estimation by the RANSAC algorithm has better robustness than others. The main idea of the RANSAC algorithm is that the initial values of objective plane function parameters are estimated by iterative method based on extracting an appropriate amount of data points; according to these initial parameter values, points which are satisfactory estimated parameters will be separated as internal points, others as external points; then, we re-estimate parameter values by using an interior point until a minimum error is acquired. Because traffic surveillance videos are captured by cameras with short focal length, the traffic scenes mainly contain abandoned objects and the road area. Hence, to simplify road plane extraction based on an actual traffic scene, two road constraint assumptions are used: the road region is almost located in the center of the image area; the camera is fixed and the road area in the image located in the same plane, which can be expressed with a plane equation Ax+By+Cz+D=0. Sample points are selected randomly by using a normal distribution so that most of sample points are in the road region of the image center. This will enable selection of good sample points. The more the good samples are, the better d and the more reliable the road plane equation is.

(2) Height calculation of hazardous abandoned objects

Suppose there are points P=(x,y,z) and planes Ax+By+Cz=1 in 3D space, then the calculation formula of distance from a 3D point to a plane is described as follows:

(21) $$d=\frac{\left|Ax+By+Cz-1\right|}{\sqrt{A^{2}+B^{2}+C^{2}}}$$

The distance from the point of a suspected abandoned object to the road plane is calculated by using Equation (21). The biggest distance which is the biggest height of suspected-abandoned object is found according to the previous distance calculation result. If the value of the biggest height is greater than some threshold T, the target is recognized as a hazardous abandoned object, otherwise, it is judged as a harmless abandoned object.

## 3. Experimental Results and Analysis

### 3.1. Preparation

**Figure 8:**
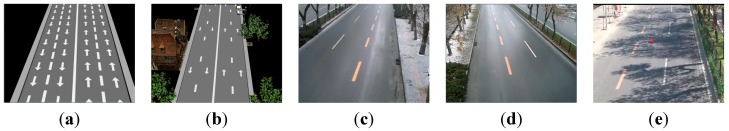
Different situation of simulations and real experiments. (**a**,**b**) simulation situation; (**c**–**e**) real road experiment situation.

**Figure 9:**
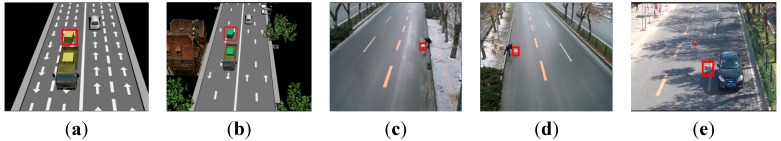
Processes of discarding abandoned objects. (**a**,**b**) are simulation examples; (**c**–**e**) are real road experiments.

In order to verify the abandoned object detection system based on three-dimensional image information can perform with high accuracy and detect hazardous objects effectively, many related simulations and experiments have been designed. All experiments were carried out on an AMD Sempron processor with 2G RAM. Algorithms are implemented on the Visual Studio 6.0 platform with OPENCV and OpenGL. We made two simulation videos with different backgrounds for abandoned objects detection using 3D MAX software. Both videos contain normal driving process, abandon process, and avoid process. To prepare for the experiments, in order to test the performance of the proposed system, we captured video from overpasses at different locations in an urban area (Beijing, China). The verification videos were in 320 × 240 resolution and a frame rate of 25 frame/s. In addition, boxes, stones, bags, bricks, and a bucket were used as abandoned objects in these experiments. The size of the object ROI was determined according to the object segmentation size. Examples of different simulation situations and real experiments are shown in Figure 8. The processes of discarding an abandoned object are shown in Figure 9, and the suspected abandoned objects are manually marked by rectangle. As Figure 8 and Figure 9 show, the background of (b) is more complex than (a), and the height of abandoned boxes, which fall off the truck, are both set as 18 cm. Abandoned objects of (c) and (d) are thrown by the pedestrian, while (e) is thrown from a car.

### 3.2. Segmentation-Performance Verification

**Figure 10:**
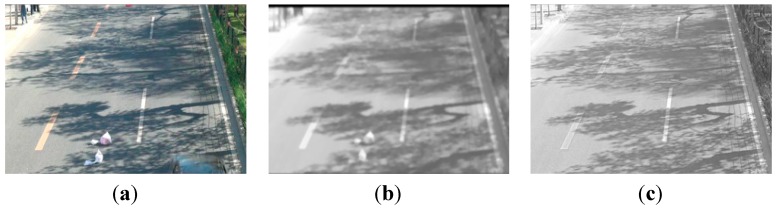
Dual-background model updating. (**a**) Frame with abandoned objects; (**b**) Short-term background; and (**c**) Long-term background.

In these experiments, three abandoned objects are placed in the road, and they are close to each other. One of the original images is shown in Figure 10a. Long and short-term background model are built and updated to adapt to real-time environmental changes. Because the learning rate of the short-term background is bigger, abandoned objects are updated into the background model after the abandoned objects are left in a traffic scene for a while, as shown in Figure 10b. The learning rate of the long-term background model makes the background not contain abandoned objects at a corresponding time as shown in Figure 10c, so the corresponding short-term background model contains the abandoned object, but the long-term background does not contain abandoned objects.

**Figure 11:**
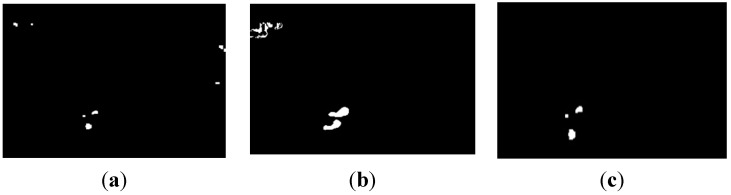
Suspected-abandoned object segmentation. (**a**) Dual-background segmentation image; (**b**) Dual-foreground segmentation image; and (**c**) Static foreground region image based on proposed dual-background difference algorithm.

In order to demonstrate the advantages of the static foreground region segmentation method described above, our suspected abandoned object segmentation results based on dual-background difference algorithm are compared with traditional methods, dual-background segmentation and dual-foreground segmentation. The detection results of Figure 10a are shown in Figure 11.

Comparing Figure 11a–c, one can observe that our static foreground region image based on the dual-background difference algorithm obtains the best segmentation result. Due to the fact the dual-background model is affected by slow-moving objects which may be categorized as background, the dual-background segmentation method exacted many more suspected regions (such as the upper-left and right of Figure 11a) than actual suspected object regions. Meanwhile, because of the deviation region between the long-term foreground and short-term foreground, the dual-foreground segmentation method obtains both suspected abandoned objects and the outline of moving objects (such as the upper-left of Figure 11b). In addition, when the color information of an object is similar to the road color or its shape characteristics are too small, different object regions may be merged into a single region by traditional methods, which will cause false abandoned object detection results. In order to solve these problems in existing methods, AND operation and morphological filtering are used in the proposed method to eliminate the disturbing information.

**Table 1:** Average abandoned object segmentation performance.

		Box	Bag	Stone	Brick	Bucket	AVERAGE
Proposed Method	Segmentation rate * (%)	92.5	87.4	97.2	94.5	90.3	92.38
	Segmentation speed ** (s)	3.11	3.38	2.83	3.22	3.00	3.108
Dual-background segmentation	Segmentation rate (%)	84.5	78.7	83.2	88.8	81.2	83.28
	Segmentation speed (s)	2.88	3.16	2.77	3.06	2.77	2.928

* $Detection\;rate=\frac{Total\;frames\;of\;correct\;segmentation\;}{Total\;frames\;of\;abandoned\;objects\;}$; ** $Detection\;speed=\frac{Frames\;number\;of\;first\;segmentation\;-\;Frames\;number\;of\;abandoned\;objects\;}{video\;FPS\;}$.

Figure 11c shows that the suspected abandoned objects are accurately and integrally segmented by the proposed dual-background difference algorithm. In order to compare the performance of the proposed algorithm with other methods, two key parameters, segmentation rate and segmentation speed, are listed in Table 1. As seen from Table 1, using dual-background segmentation makes the detection speed a little higher than our static foreground region image based on dual-background difference algorithm, but it decreases the average detection rate nearly 10%. Table 1 indicates that the proposed segmentation method can achieve a good performance and meets the requirement of fast detection.

### 3.3. 3D Reconstruction and Recognition Performance Verification

After detecting a suspected abandoned object, the camera internal parameters are calculated using Zhang’s camera calibration method. Then, an image of the abandoned object is captured to get matching points that are used to estimate the fundamental matrix, essential matrix, rotation matrix R and translation vector T by part of the BIRR algorithm.

**Figure 12:**
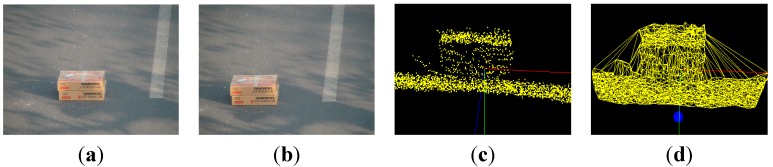
3D object reconstruction result of boxes. (**a**) left scene; (**b**) right scene; (**c**) point cloud; and (**d**) reconstruction result of boxes.

**Figure 13:**
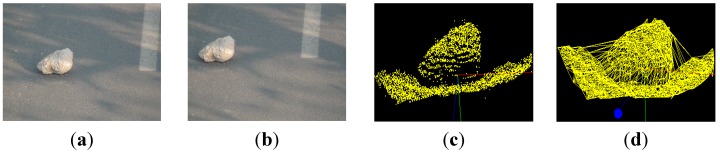
3D object reconstruction result of stone. (**a**) left scene; (**b**) right scene; (**c**) point cloud; and (**d**) reconstruction result of stone.

If the coordinates of the left camera A are defined as the world coordinate system, then the coordinates of position B can be acquired based on a rotation matrix R and translation vector T. Then, according to the perspective projection relationship between image coordinate system and the world coordinate system, 3D coordinate values of feature points are calculated by least squares approximation. The reconstruction result of the box and stone are shown in Figure 12 and Figure 13, respectively. Images (a) and (b) are the scenes of the binocular camera. (c) is the point cloud data of objects. Image (d) shows the reconstruction result of objects.

**Figure 14:**
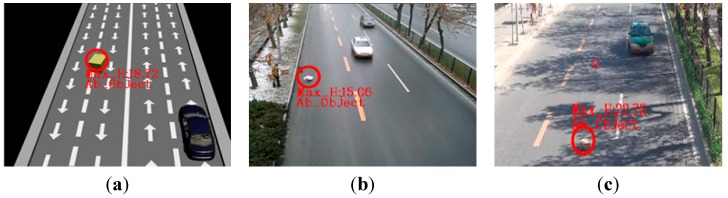
Abandoned object detection for different scenarios. (**a**) detection result in simulations situation; (**b**,**c**) detection results in real road experiments situation.

The final goal of an abandoned object detection system is that it provide specific alarm information about dangerous abandoned objects to relevant administrative departments. The safety threshold for the system alarm is set as 10 cm, according to the height of a normal automobile chassis. In order to verify the overall algorithm can meet the requirements, the height calculation algorithm and its alarm are verified through numerous experiments. Experimental results of abandoned objects are shown in Figure 14. In these figures, the maximum heights of the objects are 18.72 cm, 15.06 cm, and 22.75 cm, respectively. Therefore, they are detected and marked as dangerous abandoned objects.

**Figure 15:**
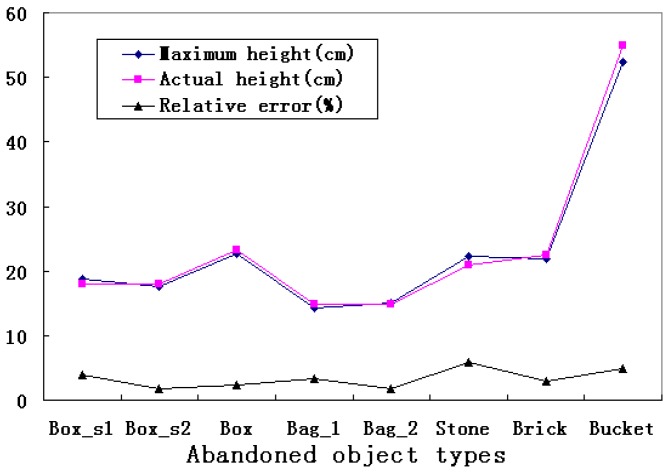
Results of abandoned object detection system.

Our method successfully reconstructs all the objects and road plane, and can give good results for calculating the height of abandoned object. In order to verify the validity of the calculation theory of target maximum height, this paper calculated the target maximum height by each point of target on the basis of calculating road plane equation. Relative error was used to estimate the accuracy of 3D reconstruction of abandoned objects. The experimental results are shown in Table 2 and Figure 15.

**Table 2:** Analysis of height calculation.

	Maximum Height (cm)	Actual Height (cm)	Relative Error * (%)
Box_s1	18.72	18.00	4.00
Box_s2	17.68	18.00	1.78
Box	22.75	23.30	2.36
Bag_1	14.30	14.80	3.38
Bag_2	15.06	14.80	1.76
Stone	22.24	21.00	5.90
Brick	21.82	22.50	3.02
Bucket	52.32	55.00	4.87

* $\text{Relative error}=\frac{\left|\text{Maximum height-Actual height}\right|}{\text{Actual height}}\times 100\text{\%}$.

Box_s1 and Box_s2 represent the abandoned objects of simulation. Bag_1 and Bag_2 represent the same object type in different experiments. Experimental results show that the calculated height is close to the actual height. The relative error is about 3.38% on average and that the maximum error is less than 6%. It indicates that our system has stabilization errors and provides an acceptable performance.

## 4. Conclusions

Road traffic surveillance is a complex circulation system of people, vehicles and the road environment. Fast and effective detection of unexpected abandoned objects is very important to prevent traffic accident and avoiding property losses. In most of the existing methods, abandoned objects are detected by a single camera. However, with these methods it is difficult to obtain the objects’ heights, which is one of the key features to judge the danger posed by an abandoned object. This leads to low hazardous abandoned objects detection precision. In this paper, a new Binocular Information Reconstruction and Recognition (BIRR) algorithm is proposed to imping abandoned objects for road traffic surveillance. In detail, a static foreground region image segmentation algorithm is proposed to calculate suspected abandoned object, which lays a solid foundation for further processing. 3D information of suspected abandoned objects is reconstructed by the proposed theory. Hazardous abandoned objects are recognized by the heights of the suspected abandoned objects. The experimental results demonstrated the successful recognition of different abandoned objects. The abandoned objects detection system can be used for fast traffic surveillance systems. Although the proposed system exhibited acceptable performance, further studies are needed to improve the speed of the proposed algorithms using a Digital Signal Processor (DSP).
